# Long-term clinical outcomes of Ahmed valve implantation in patients with refractory glaucoma

**DOI:** 10.1371/journal.pone.0187533

**Published:** 2017-11-02

**Authors:** Chang Kyu Lee, Kyoung Tak Ma, Young Jae Hong, Chan Yun Kim

**Affiliations:** 1 Department of Ophthalmology, Ulsan University Hospital, University of Ulsan College of Medicine, Ulsan, Korea; 2 Jeil Eye Clinic, Soowon, Korea; 3 Nune Eye Hospital, Seoul, Korea; 4 Institute of Vision Research, Department of Ophthalmology, Yonsei University College of Medicine, Seoul, Korea; Boston University School of Medicine, UNITED STATES

## Abstract

**Purpose:**

To evaluate the long-term efficacy of intraocular pressure (IOP) reduction and complications of Ahmed Glaucoma Valve (AGV) implantation in patients with refractory glaucoma.

**Design:**

Retrospective study.

**Subjects:**

The study involved 302 refractory glaucoma patients who underwent AGV implantation and had a minimum follow-up of 6 months between March 1995 and December 2013.

**Methods:**

An operation was defined as successful when (1) the postoperative IOP remained between 5 and 21 mmHg and was reduced 30% compared to the baseline IOP with or without medication, (2) there was no loss of light perception or vision-threatening severe complications, and (3) no additional filtering or aqueous drainage surgery was required. Clinical records were reviewed.

**Main outcome measures:**

IOP, anti-glaucoma medications, and complications

**Results:**

The mean follow-up period was 62.25 months (range, 6 to 190 months). The cumulative probability of success was 89% at 6 months, 81% at 1 year, 66% at 3 years, 44% at 10 years, and 26% at 15 years. IOP was reduced from a mean of 32.2 ± 10.5 mmHg to 18.6 ± 9.1 mmHg at 1 month, 15.2 ± 7.0 mmHg at 6 months, and 14.2 ± 3.5 mmHg at 15 years. Surgical failures were significantly increased when preoperative IOP was high, and when severe complications occurred after AGV implantation (P < 0.05).

**Conclusion:**

AGV implantation was successful for IOP control in patients with refractive glaucoma in the long term. However, the success rate of surgery decreased over time. Preoperative high IOP and severe complications related to the operation were significant risk factors for failure.

## Introduction

Refractory glaucoma such as neovascular, uveitic, and angle recession glaucoma, cases of previously failed glaucoma surgery, and other secondary glaucomas are usually known as poorly responsive to traditional glaucoma medical and surgical procedures [1–3]. If medical and surgical therapy were not effective, diverse surgical approaches have been proposed for the treatment of refractory glaucomas. These include trabeculectomy with mitomycin and cyclophotocoagulation procedures. [2, 4–6]The use of glaucoma drainage devices (GDDs) is reserved also for cases of glaucoma not adequately controlled by conventional treatment modalities or in patients with high-risk disease, such as those with refractory glaucoma [7, 8]. The Tube Versus Trabeculectomy study demonstrated that in patients who had undergone previous intraocular surgery, glaucoma drainage devices had a higher success rate with fewer complications when compared to trabeculectomy with mitomycin C after 3 years of follow- up [9]. The Ahmed Glaucoma Valve (AGV; New World Medical, Inc.; Rancho Cucamonga, California, USA) is a venture-based, flow-restrictive valve designed to reduced postoperative hypotony and its complications [10, 11]. However, there have been reports of high rates of encapsulation and ocular hypertension associated with this device, as well as an increased requirement for postoperative glaucoma medications [10–14]. Moreover, the overall success rate of Ahmed valve implantation ranges from 63% to 100% at one year, while the maximal long-term follow-up success rate has been reported to be 49% at five years [6]. Very long-term follow-up data are scarce and studies on severe complications, especially those affecting clinical outcomes, have rarely been presented thus far. We thus set out to evaluate the very long-term clinical outcomes of Ahmed valve implantation in refractory glaucoma, including its severe complications and success rate.

## Patients and methods

This study adhered to the tenets of the Declaration of Helsinki and was approved by our institutional review board(IRB). IRB/Ethics committee of yonsei university health system approval was obtained(approval number:4-2013-0108). We conducted a detailed retrospective review of the records of patients who had undergone Ahmed glaucoma valve implantation performed by two surgeons (YJH and CYK) between March 1995 and December 2013 at Severance Hospital in Seoul, Korea. Moreover data of patients was accessed anonymously(S1 Table), the need for informed consent was waived by our IRB. Patients were excluded if they had previously undergone aqueous shunt implantation in the study eye at the time of the study or had not yet completed six months of follow-up at the time of record review. In this study, operations on eyes that had already been trabeculetomized before the study period and then underwent Ahmed valve implantation were classified as secondary operations, while operations on eyes that underwent Ahmed valve implantation as a first glaucoma surgery during the research period were classified as primary operations. The rates of success were compared between primary and secondary operations. Indications for surgery included uncontrolled IOP despite maximal topical therapy, previously failed conventional glaucoma surgery, or both. We collected preoperative data from the records of the patients. We analyzed age at the time of surgery, sex, eye laterality, visual acuity, IOP, prior ocular surgery, specific glaucoma diagnosis and other ocular history, number of medications, and 24–2 full-threshold or Swedish interactive threshold algorithm visual field mean deviation (MD).

Postoperative data were collected from the records of patients from all consecutive visits. Data collected included IOP, number of medications used, additional surgeries performed, follow-up time, and surgical complications classified as mild or severe and led to loss of visual acuity. These data were collected at one, three, 6, 12 (one year), 36 (three years), 60 (five years), 120 (ten years), and 180 (fifteen years) months after surgery.

### Surgical technique

The intraoperative sequences of the surgical procedures varied slightly from case to case according to the surgical history and clinical status of each eye and the discretion of the treating surgeons. Polypropylene and silicone AGVs (models S-2 and FP-7, respectively) were implanted by two surgeons (YJH and CYK). A fornix-based flap of the conjunctiva and Tenon’s capsule was created in the superior temporal or superior nasal quadrant. The AGVs were positioned in the middle of the quadrant, with the anterior edge of the plate 10 mm posterior to the superior temporal corneoscleral limbus or 8 mm posterior to the superior nasal corneoscleral limbus. The tube was flushed with balanced salt solution to ensure patency before insertion and was trimmed to the correct length with a beveled edge. A paracentesis was created using a 23-gauge needle at the limbus parallel to the iris. Viscoelastic was injected to maintain the anterior chamber before tube insertion. The tube was covered with a commercially available processed pericardial graft (Tutoplast; IOP, Inc.; Costa Mesa, California, USA) or partial-thickness self-scleral flap. The conjunctiva was closed using 10–0 nylon or 8–0 vicryl, depending on surgeon preference. After the operation, topical antibiotics and topical steroids were used for 4 or 6 weeks.

### Criteria for success

An operation was considered successful when (1) the postoperative IOP remained between 5 and 21 mmHg and was reduced 30% from the baseline IOP with or without medication, (2) there was no loss of light perception after surgery or vision-threatening severe complications, and (3) no additional filtering surgery or aqueous drainage surgery was required one, 3, 6, 12, 36, 60, 120, or 180 months after surgery.

### Statistical methods

Kaplan-Meier survival analyses were used to assess long-term success rates according to the criteria defined above. Cox proportional hazard regressions were used to evaluate the effects of baseline clinical characteristics on success. Categorical data were compared using the chi-square test. Log rank analysis was used to compare success rates of primary and secondary operations. All analyses were conducted using the Statistical Package for the Social Sciences 20.0 for Windows (SPSS, Chicago, IL, USA). P values less than 0.05 were considered statistically significant.

## Results

During the research period, 352 patients with refractory glaucoma underwent AGV implantation. We considered the eye with the more severe glaucoma included if the patient had undergone the AGV procedure in both eyes. Three-hundred and two eyes from 302 patients with refractory glaucoma were included in the study after considering the inclusion and exclusion criteria. The mean (standard deviation [SD]) age of the 302 eyes was 52.31 (18.40) years and the mean postsurgical follow-up time was 62.25 months (range, 6–190 months). The mean MD value of the preoperative visual field test was -17.54 dB, and the mean (±SD) preoperative visual acuity was 1.54 logarithm of the minimum angle of resolution (logMAR) units (±1.12 logMAR units).

The most common type of refractory glaucoma requiring AGV implantation was neovascular glaucoma (39.1%), followed by secondary glaucoma (32.1%), primary open angle glaucoma (16.6%), congenital glaucoma (6.6%), and primary angle closure glaucoma (5.0%). The most common type of secondary glaucoma was uveitis (43%), followed by glaucoma after non-glaucoma intraocular surgery (37.2%), and iridocorneal endothelial syndrome (10.5%) (Table 1).

**Table 1:** Preoperative demographic characteristics of the patients (n = 302).

Age (y)	
Mean ± SD	52.31 ± 18.40
Range	1–85
Sex	
Male/Female	198/104
Laterality	
Right eye/Left eye	150/152
Length of follow-up (months)	
Mean ± SD	62.25 ± 37.15
Range	6–190
Visual acuity (logMAR) (mean ± SD)	1.54 ± 1.12
Visual field MD (mean ± SD)	-17.54 ± 10.54
Preoperative IOP (mmHg)	
Mean ± SD	32.23 ± 10.56
Range	18–66
No. of preoperative medications	
Mean ± SD	3.4 ± 0.9
Range	1–4
Primary: Secondary	200:102
Diagnosis (%)	
POAG	50 (16.6)
PACG	15(5.0)
NVG	120(39.1)
Congenital glaucoma	20(6.6)
Secondary glaucoma	97(32.1)
Uveitis(%)	43
Glaucoma after intraocular surgery(%)	37.2
Trauma(%)	5.8
Steroid-induced glaucoma(%)	3.4
ICE syndrome(%)	10.5

SD: standard deviation, MD: mean deviation, POAG: primary open angle glaucoma,

Primary: primary operation, Secondary: secondary operation, PACG: primary angle closure glaucoma, NVG: neovascular glaucoma, ICE syndrome: iridocorneal endothelial syndrome

The numbers of eyes at the different postoperative follow-up time points were 302 (100%) at 6 months, 286 (94.7%) at 12 months, 160 (53%) at 36 months, 102 (33.7%) at 60 months, 68 (22.5%) at 120 months, and 11 (3.6%) at 180 months after surgery.

**Fig 1:**
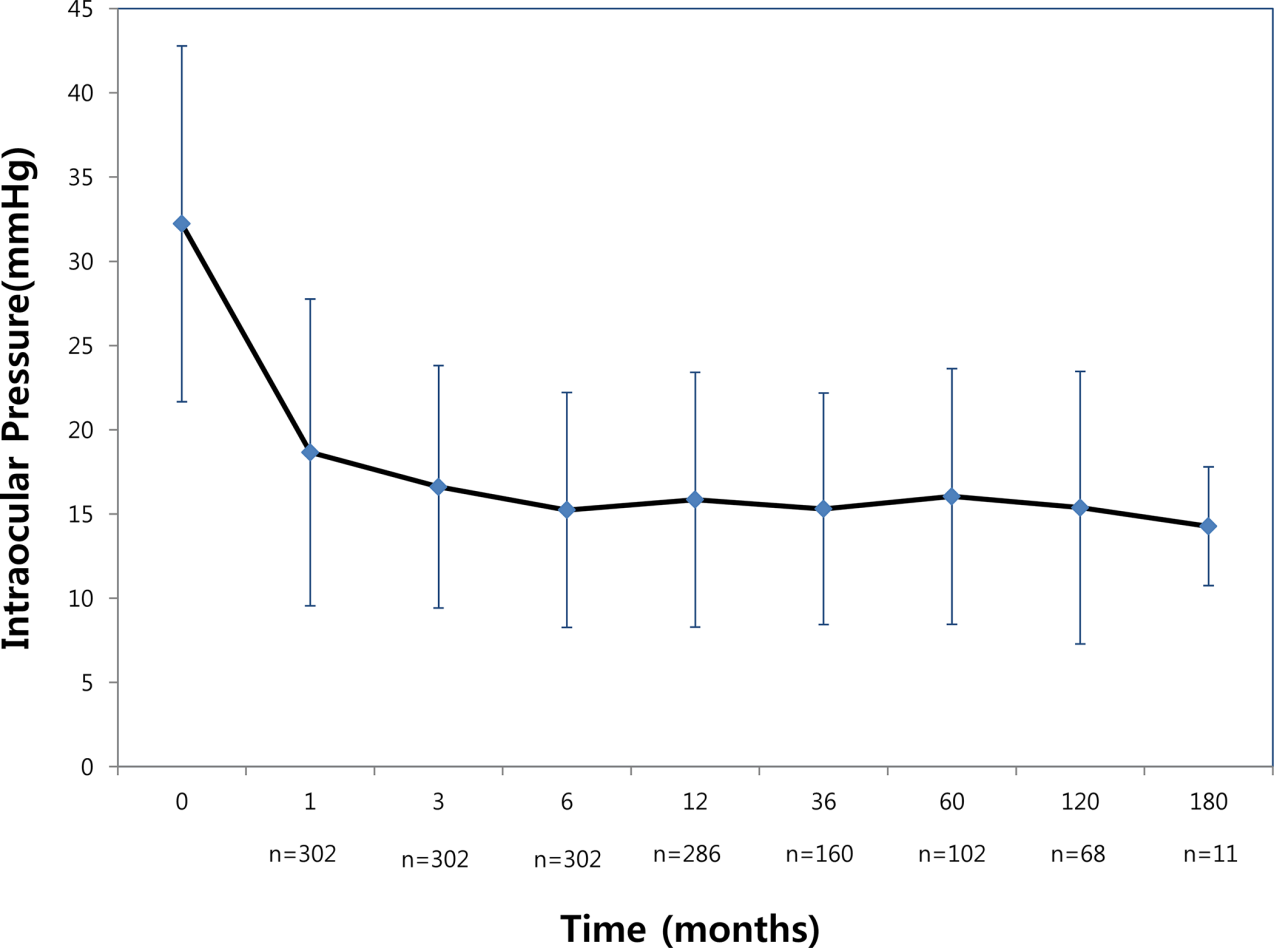
Mean intraocular pressure(IOP) before surgery and during follow-up. The intervening mean postoperative IOPs were significantly reduced compared to baseline during entire postoperative periods. Error bars indicate 1 standard deviation.

The mean IOP (±SD) before AGV implantation was 32.2 ± 10.6 mmHg (range, 18–66 mmHg). This was reduced to 18.6 ± 9.1 mmHg at 1 month, 15.2 ± 6.9 mmHg at 6 months, 15.3 ± 8 mmHg at 120 months, and 14.2 ± 3.5 mmHg at 180 months. The intervening mean postoperative IOPs ranged from 13 mmHg to 18 mmHg during the entire postoperative period, and were significantly reduced compared to baseline (p < 0.05) (Fig 1).

**Fig 2:**
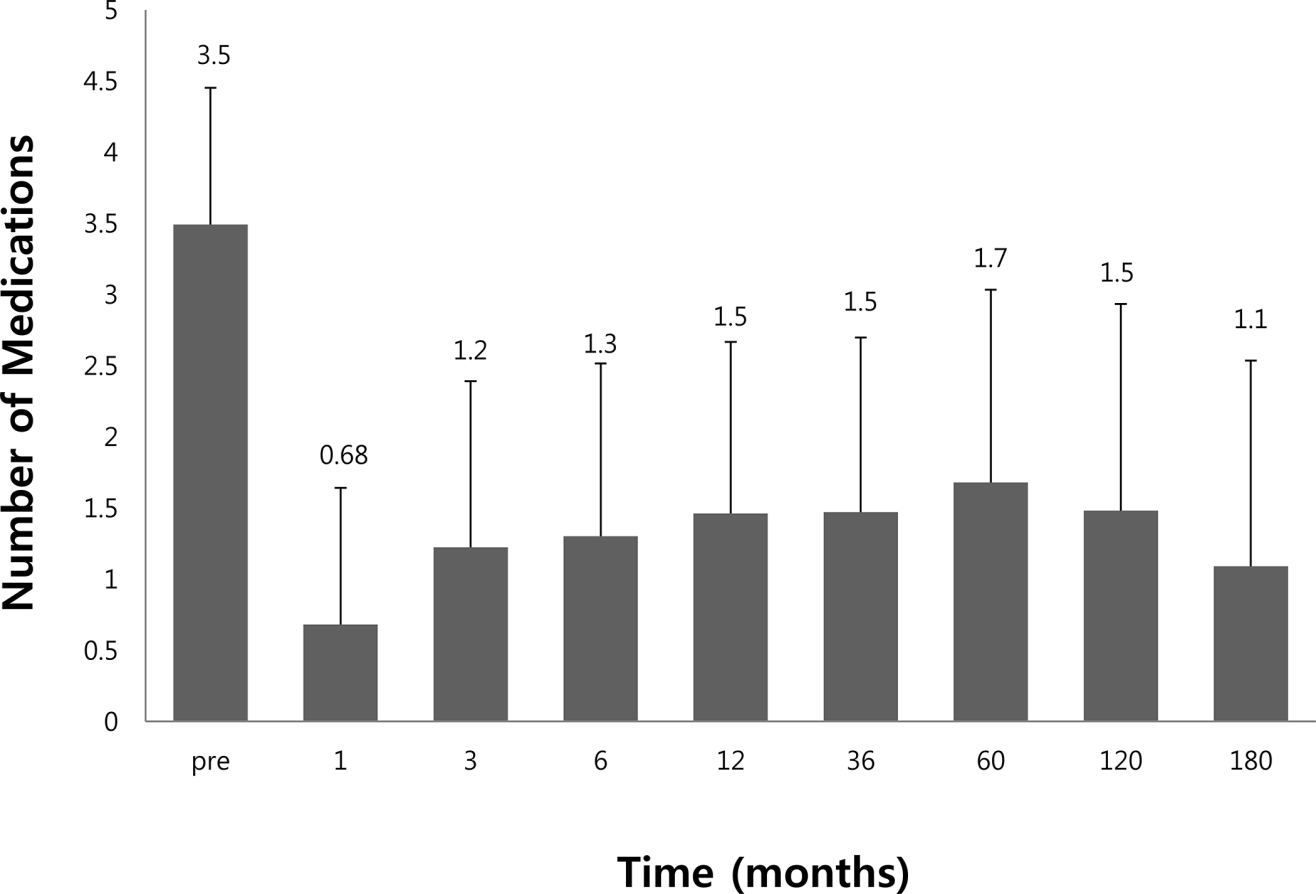
Mean number of medications before surgery and during follow-up. The mean number of postoperative glaucoma medications ranged from 0.6 to 1.7 during the entire postoperative period. Error bars indicate 1 standard deviation.

The mean (±SD) number of glaucoma medications decreased from 3.5 ± 0.9 medications at baseline to 0.7 ± 0.9 at 1 month, 1.2 ± 1.1 at 3 months, 1.5 ± 1.2 at 12 months, 1.5 ± 1.4 at 120 months, and 1.1 ± 1.4 mmHg at 180 months. The mean number of postoperative glaucoma medications ranged from 0.6 to 1.7 during the entire postoperative period (Fig 2).

The prevalence of postoperative complications was 25% (76 eyes). These complications included tube problems, such as tube exposure, tube obstruction, tube migration, and tube-corneal touching, which occurred in 24 eyes (8%), and were the most common postoperative complications. Mild to moderate hyphema was observed in 15 eyes (5%). All of the hyphemas were absorbed spontaneously. Transient hypotony, defined as IOP of less than 5 mmHg, occurred in 11 eyes (4%). One eye (0.3%) had diplopia due to AGV implantation. Previous complications were classified as mild because they were resolved spontaneously and did not threaten visual acuity in the long term. However, severe complications that threatened visual acuity included endophthalmitis, corneal decompression, and phthisis. Phthisis occurred in 15 eyes (5%), corneal decompression affected 7 eyes (2.3%), and endophthalmitis was observed in 3 eyes (1%) (Table 2).

**Table 2:** Postoperative complications for AGV implantation (n = 76).

Complications	Cases(%)
Mild	
Hypotony	11(3.6)
Tube problems	24(7.9)
Hyphema	15(5)
Diplopia	1(0.3)
Severe	
Corneal decompression	7(2.3)
Phthisis	15(5)
Endophthalmitis	3(1)

Severe complications were significantly different according to age at the time of operation and increased at old age (p = 0.04). In addition, preoperative uveitis glaucoma was associated with significantly higher numbers of severe complications (Table 3).

**Table 3:** Several factors related to severe complications.

	Severe Complications(cases)	Mild Complications(cases)	*P-*value*
Age class(years)			0.044
1–20	2	2	
21–40	6	1	
41–60	12	21	
61–90	24	5	
Secondary Glaucoma			0.001
Uveitis	9	4	
Glaucoma after surgery	2	9	
Trauma	1	1	
SIG	0	0	
ICE syndrome	2	1	

*P-values are based on chi-square tests.

SIG: steroid-induced glaucoma, ICE syndrome: iridocorneal endothelial syndrome

Fifty eyes (16.5%) required additional glaucoma surgery, such as trabeculectomy and AGV implantation, during the entire follow-up period. Twenty-four eyes underwent trabeculectomy and twenty-six eyes underwent additional AGV implantation.

**Fig 3:**
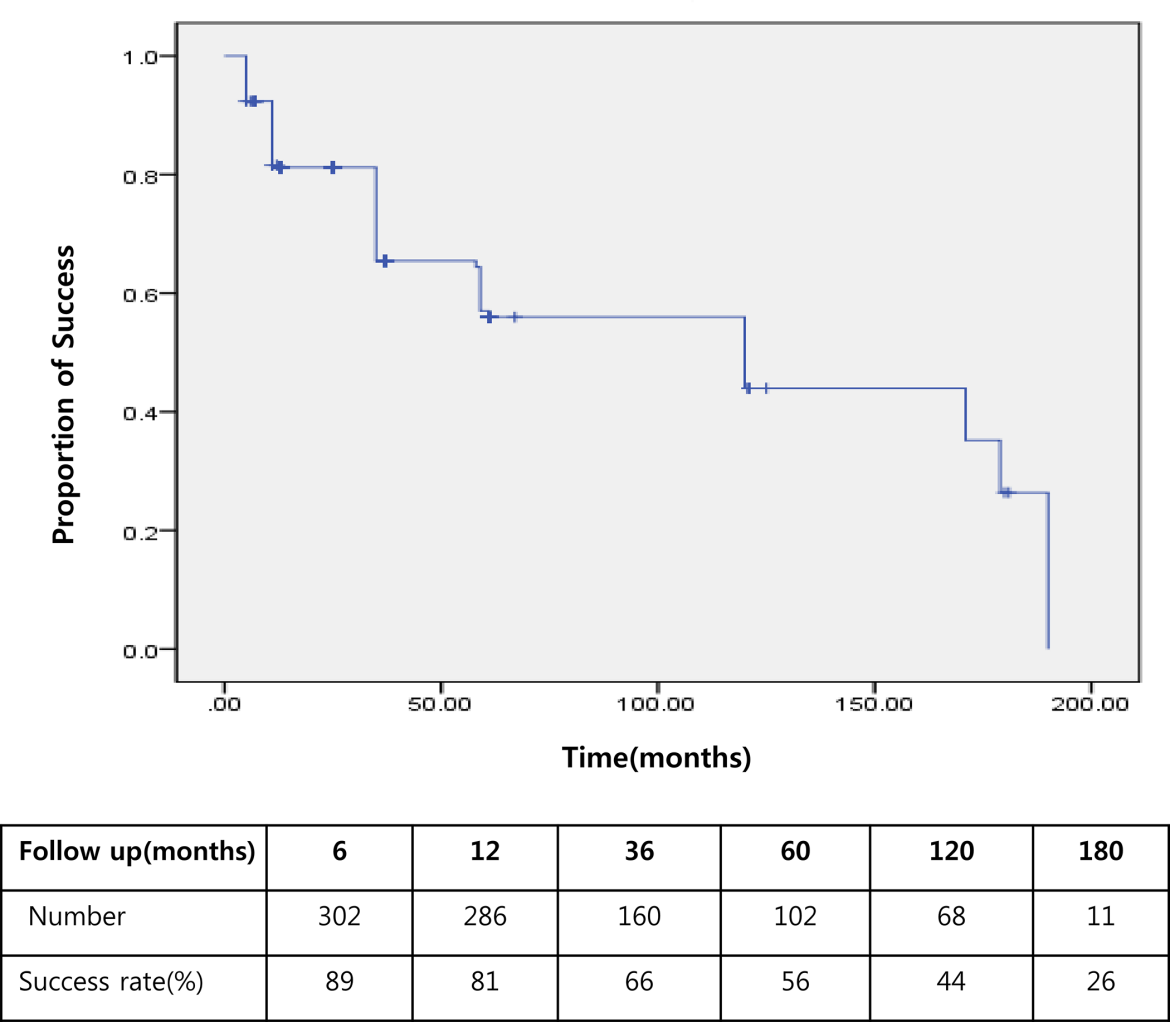
Kaplan-Meier estimates of the cumulative probability of valve success for AGV implantation. The success rate for patients with refractory glaucoma who had undergone AGV implantation was decreased with time. Percent of success cases and number of eyes during the follow-up period are shown in the box.

Kaplan-Meier estimates of the cumulative probability of valve success for AGV implantation are plotted in Fig 3.

**Fig 4:**
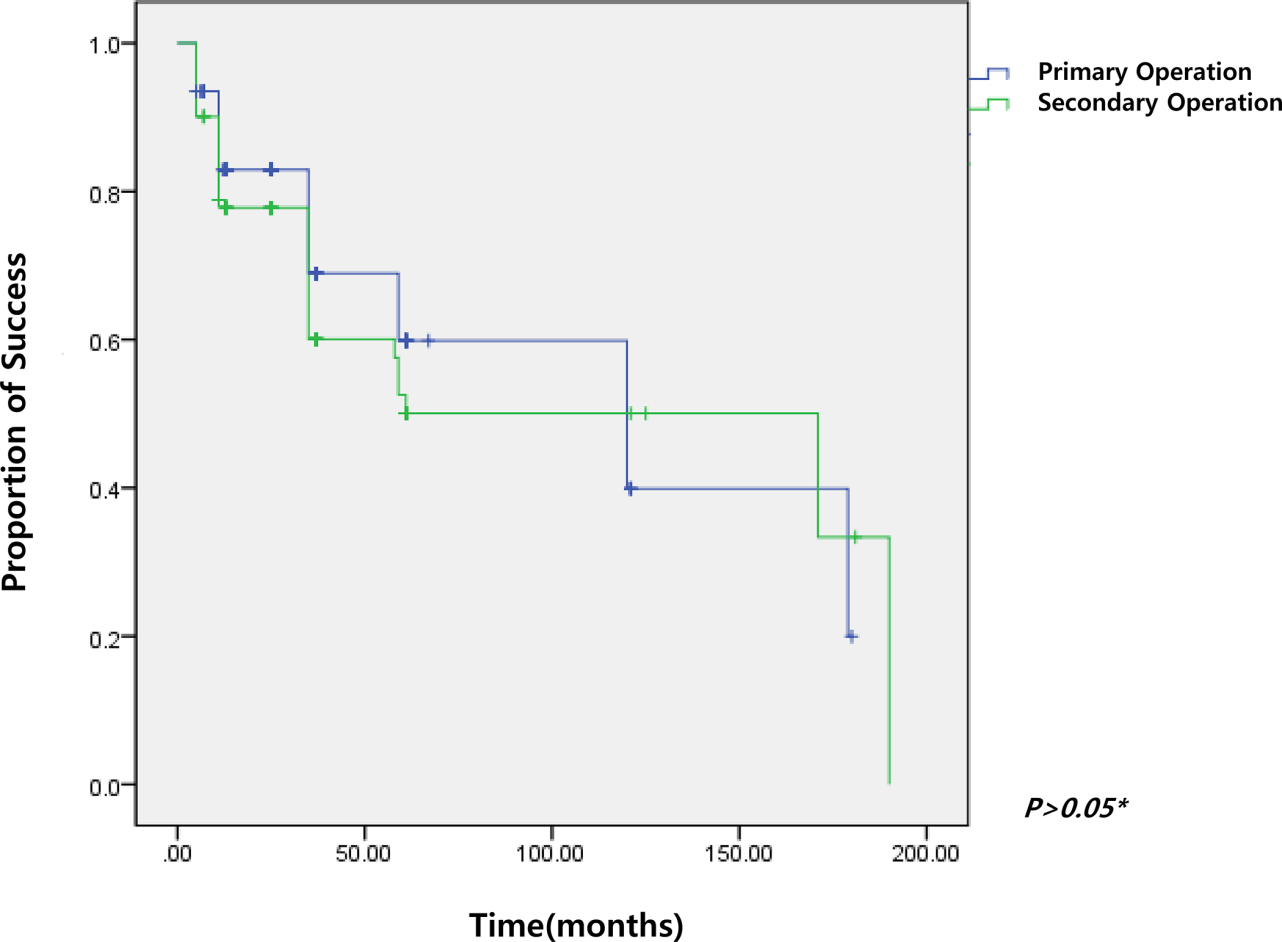
Kaplan-Meier cumulative probability curve of success in patients with primary and secondary surgery. The success rates after surgery were not significantly different between primary AGV implantation and secondary AGV implantation. P* value was calculated using log rank analysis.

There was 89% success rate at 6 months and 81%, 66%, 56%, 44%, and 26% success rates at 12, 36, 60,120, 180 months, respectively. The success rates after surgery were not significantly different between primary AGV implantation and secondary AGV implantation (p > 0.05) (Fig 4).

The Cox proportional hazard model indicated that eyes with preoperative high IOP were at increased risk for failure (p = 0.029). Several severe postoperative complications were also significantly associated with an increased risk for failure (p = 0.004) (Table 4).

**Table 4:** Risk factors for surgical failure in patients with refractory glaucoma who underwent Ahmed valve implantation, and results from Cox proportional hazard regression models.

Characteristic	RR	95%CI	*P*-value
Age(y)	0.99	0.85 to 1.25	0.88
Preoperative IOP	1.2	1.02 to 3.54	0.029^§^
Mild Complications	0.87	0.65 to 2.34	0.87
Severe Complications	1.83	1.65 to 4.25	0.004^§^
Total FU	0.99	0.78 to 2.82	0.42
Preoperative Diagnosis	0.95	0.47 to 4.57	0.69

^§^Significant difference, *P* < 0.05

RR: relative risk, FU: follow-up

## Discussion

Patients with refractory glaucoma are unresponsive to routine treatment, including medical treatment for lowering IOP and traditional surgical procedures. Most cases of refractory glaucoma consist of secondary glaucoma with complex features, including very high IOP, various and/or unknown mechanisms for elevated IOP, limited assessment and treatment due to perplexing ocular factors, the need for combination therapies, poor prognosis, and rapid deterioration in vision [14, 15]. Traditional surgical management for refractory glaucoma includes trabeculectomy as the first choice, while GDDs are reserved for patients with intractable glaucoma. However, recent studies have shown GDDs to have success rates comparable to trabeculectomy with antimetabolite therapy [9, 16, 17].

AGV was launched in 1993 as the first GDD with a unidirectional valve mechanism contributing to the prevention of postoperative hypotension [10]. Currently, there are two different models of AGV with different surface areas: FP8 (96 mm^2^) is used in children, and FP7 (184 mm^2^) is usually used in adults. Numerous retrospective studies have produced long-term (5–6 years) clinical data on the use of AGV implantation in various types of intractable glaucoma [6, 14, 17]. Here we present very long–term (maximum of 190 months) follow-up clinical results on patients with refractory glaucoma with Ahmed glaucoma valve implants.

In our study, preoperative IOP was 32.2 ± 10.5 mmHg, postoperative IOP was 18.6 ± 7.1 mmHg at 1 month, 16.6 ± 7.1 mmHg at 3 months, and 15.2 ± 6.9 mmHg at 6 months. IOP was maintained at 14–16 mmHg for the remaining period. We found out that IOP was relatively high until 3 months after the operation, and was then steady afterward. The mean number (±SD) of preoperative glaucoma medications was 3.5 ± 0.9, while this number was reduced to 0.6 ± 0.9 at 1 month, 1.2 ± 1.2 at 3 months, 1.3–1.6 afterward. Therefore, our study demonstrates that as IOP increases during the 1–3 months following the operation, the mean number of glaucoma medications increases to 1.2 during the early postoperative period. This increase in the number of medications seems to be correlated to the hypertensive phase, which is a term that refers to a period of transient elevation of IOP after glaucoma drainage implant surgery [14, 18]. Nouri-Mahdavi and Caproli describe an early “hypertensive phase “that occurs when the IOP increases above 21 mmHg after an initial postoperative IOP reduction to 21 mmHg or less [12]. This phase was observed in 15.8 and 18.4% of patients in the AGV group at 3 and 6 months after surgery, respectively. This is a common observation following Ahmed valve implantation, and has incidences ranging from 40 to 80% [18].

Tube problems, which included tube exposure, tube obstructions, tube migration, and tube-corneal touching, were the most common mild complications in the postoperative period. Ou et al. have reported that tube-related problem are the most common complications after AGV implantation in patients with primary congenital glaucoma, and that most of the tube-related problems are due to tube-corneal touching [19]. Tube exposures are also a major tube problem. Tube exposure leads to inflammation-mediated melting of self-tissue or the donor graft, and subsequent mechanical damage to the overlying conjunctiva lying at the heart of the protrusion by the underlying valve tube [20]. These tube-related problems are highly correlated to severe postoperative complications that affect visual acuity, as shown in previous studies [21–23]. It is well-known that untreated cases of tube exposure may lead endophthalmitis with poor prognosis. Morad et al. reported three cases of endophthalmitis after AGV implantation, two of which were associated with tube exposure and subsequent infection [21]. Moreover, tube-corneal touching is well-known to be highly related to corneal complications. Topouzis et al. hypothesized that previous ocular surgeries, inflammation, and transient tube-endothelial contact may decrease endothelial cell counts and predispose patients to corneal complications [22]. Lee et al. also investigated the rate of change in endothelial cell numbers for 24 months after AGV implantation. The average numbers of endothelial cells lost after AGV implantation were 5.8% within 1 month, 11.5% after 6 months, 15.3% after 12 months, 16.6% after 18 months, and 18.6% after 24 months. The greatest loss of endothelial cells was 22.6%, and was observed in the area of the valve tube. Cell loss in the center area of the cornea was only 15.4% even 24 months after surgery [23].

Endophthalmitis, corneal decompression, and phthisis, which may be induced by tube problems were considered severe complications, as they may affect postoperative visual acuity. These severe complications were significantly more prevalent at older ages (ages of 61–90 years), and in patients with uveitis with secondary glaucoma. Therefore, old age and secondary glaucoma, especially that in patients with uveitis, lead to higher risk for tube problems. Earlier treatment of tube problems may thus prevent severe postoperative complications, such as endophthalmitis, corneal decompression, and phthisis.

Kaplan-Meier analysis indicated that the success rates of refractory glaucoma were 82%, 81%, 66%, 56%, and 21% at 6 months, 12 months, 36 months, 120 months, and 180 months, respectively. There is often difficulty when comparing intermediate-term and long-term follow-up studies, as there is a lack of uniform success criteria. In addition, different studies may have different patient demographics. As a result, the cumulative probability of success varies widely in different studies, and ranges from 49% at five years of follow-up in a retrospective study, published by Carlos and associates,^14^ to 94% in a retrospective case series of patients with uveitic glaucoma with one year of follow-up published by DaMata and associates [24]. Our short-term (89% at one year) and middle-term (56% at 60 months) success rates were comparable to others reported in the literature with similar success criteria [6, 24]. However, our results indicate that the success rate drops at later times, and reaches 26% at 180 months. One possible explanation for this observation may involve encapsulation, which has already been discussed in a number of previous studies. The Ahmed-FP7 implant has a smaller plate area (184 mm^2^) and has been associated with higher long-term pressure secondary to excessive late fibrous encapsulation of the plate, which results in surgical failure [11, 12, 14]. Inflammation has been implicated as a cause of bleb encapsulation in previous Ahmed models using plates made of polypropylene, which induce an immune response in animal models [25–28]. Although the FP-7 model has a silicone plate, it is still hypothesized that postoperative intracameral inflammatory mediators may contribute to encapsulation, especially given the lack of early flow restriction [13, 29]. The smaller filtration area, additional resistance created by the flow-restrictive mechanism, and exposure to postoperative intracameral inflammatory mediators, place the AGV implant at higher risk of encapsulation than other glaucoma drainage devices [13, 29]. Other possible reasons for surgical failure are postoperative complications. As mentioned previously, several mild complications may lead to severe complications that directly affect vision in patients with glaucoma.

Souza et al. have shown that previous glaucoma surgery is a risk factor for failure [6]. However, there was no significant difference in cumulated success rate between primary operation and secondary operation. We can therefore postulate that previous glaucoma surgery does not affect the success rate of Ahmed valve implantation in the long term.

Limitations of our study include its retrospective non-randomized design, follow-up loss at later time points, and the use of two different types of AGV. There is also a possibility of overestimation of surgical failure, as patients with more severe pathologies may continue to visit the ophthalmologist for longer periods. The strengths of the study include the fact that all of the operations were performed at a single hospital by two surgeons using a standard AGV implantation protocol, very long-term follow-up, and the large number of cases from Korea.

In conclusion, the success rate of Ahmed valve implantation is about 89% 6 months after the operation. This procedure leads to IOP levels that are relatively steady and remain in the mid-teens. The average number of glaucoma medications was between one and two starting at three months following the surgery and lasting for the long term. However, the success rate drops as times goes on. Preoperative high IOP and severe complications were the major risk factors for failure. Because of their correlation with severe complications, tube-related problems, which were the most common complications, should be managed promptly to increase success in the long term.

## Supporting information

S1 Table The anonymous data sheet of this study. (XLSX)

## References

[1] Das JC, Chaudhuri Z, Sharma P, Bhomaj S. The Ahmed Glaucoma Valve in refractory glaucoma: experiences in Indian eyes. Eye (Lond). 2005; 19: 183–190. doi: 10.1038/sj.eye.6701447 15258600 10.1038/sj.eye.6701447

[2] Lima FE, Magacho L, Carvalho DM, Susanna R Jr., Avila MP. A prospective, comparative study between endoscopic cyclophotocoagulation and the Ahmed drainage implant in refractory glaucoma. J Glaucoma. 2004; 13: 233–237. 15118469 10.1097/00061198-200406000-00011

[3] Syed HM, Law SK, Nam SH, Li G, Caprioli J, Coleman A. Baerveldt-350 implant versus Ahmed valve for refractory glaucoma: a case-controlled comparison. J Glaucoma. 2004; 13: 38–45. 14704542 10.1097/00061198-200402000-00008

[4] Tanimoto SA, Brandt JD. Options in pediatric glaucoma after angle surgery has failed. Curr Opin Ophthalmol. 2006; 17: 132–137. doi: 10.1097/01.icu.0000193091.60185.27 16552247 10.1097/01.icu.0000193091.60185.27

[5] Mandal AK, Walton DS, John T, Jayagandan A. Mitomycin C-augmented trabeculectomy in refractory congenital glaucoma. Ophthalmology. 1997; 104: 996–1001; discussion 1002–1003. 9186441 10.1016/s0161-6420(97)30195-x

[6] Souza C, Tran DH, Loman J, Law SK, Coleman AL, Caprioli J. Long-term outcomes of Ahmed glaucoma valve implantation in refractory glaucomas. Am J Ophthalmol. 2007; 144: 893–900. doi: 10.1016/j.ajo.2007.07.035 17916318 10.1016/j.ajo.2007.07.035

[7] Schwartz K, Budenz D. Current management of glaucoma. Curr Opin Ophthalmol. 2004; 15: 119–126. 15021223 10.1097/00055735-200404000-00011

[8] Minckler DS, Francis BA, Hodapp EA, Jampel HD, Lin SC, Samples JR, et al Aqueous shunts in glaucoma: a report by the American Academy of Ophthalmology. Ophthalmology. 2008; 115: 1089–1098. doi: 10.1016/j.ophtha.2008.03.031 18519069 10.1016/j.ophtha.2008.03.031

[9] Gedde SJ, Schiffman JC, Feuer WJ, Herndon LW, Brandt JD, Budenz DL. Three-year follow-up of the tube versus trabeculectomy study. Am J Ophthalmol. 2009; 148: 670–684. doi: 10.1016/j.ajo.2009.06.018 19674729 10.1016/j.ajo.2009.06.018

[10] Coleman AL, Hill R, Wilson MR, Choplin N, Kotas-Neumann R, Tam M, et al Initial clinical experience with the Ahmed Glaucoma Valve implant. Am J Ophthalmol. 1995; 120: 23–31. 7611326 10.1016/s0002-9394(14)73755-9

[11] Huang MC, Netland PA, Coleman AL, Siegner SW, Moster MR, Hill RA. Intermediate-term clinical experience with the Ahmed Glaucoma Valve implant. Am J Ophthalmol. 1999; 127: 27–33. 9932995 10.1016/s0002-9394(98)00394-8

[12] Nouri-Mahdavi K, Caprioli J. Evaluation of the hypertensive phase after insertion of the Ahmed Glaucoma Valve. Am J Ophthalmol. 2003; 136: 1001–1008. 14644209 10.1016/s0002-9394(03)00630-5

[13] Tsai JC, Johnson CC, Dietrich MS. The Ahmed shunt versus the Baerveldt shunt for refractory glaucoma: a single-surgeon comparison of outcome. Ophthalmology. 2003; 110: 1814–1821. doi: 10.1016/S0161-6420(03)00574-8 13129882 10.1016/S0161-6420(03)00574-8

[14] Ayyala RS, Zurakowski D, Smith JA, Monshizadeh R, Netland PA, Richards DW, et al A clinical study of the Ahmed glaucoma valve implant in advanced glaucoma. Ophthalmology. 1998; 105: 1968–1976. doi: 10.1016/S0161-6420(98)91049-1 9787371 10.1016/S0161-6420(98)91049-1

[15] Zhu Y, Wei Y, Yang X, Deng S, Li Z, Li F, et al Clinical Outcomes of FP-7/8 Ahmed Glaucoma Valves in the Management of Refractory Glaucoma in the Mainland Chinese Population. PLoS One. 2015; 10: e0127658 doi: 10.1371/journal.pone.0127658 25996991 10.1371/journal.pone.0127658 PMC4440786

[16] Nguyen QH. Primary surgical management refractory glaucoma: tubes as initial surgery. Curr Opin Ophthalmol. 2009; 20: 122–125. doi: 10.1097/ICU.0b013e32831da828 19240544 10.1097/ICU.0b013e32831da828

[17] Wilson MR, Mendis U, Paliwal A, Haynatzka V. Long-term follow-up of primary glaucoma surgery with Ahmed glaucoma valve implant versus trabeculectomy. Am J Ophthalmol. 2003; 136: 464–470. 12967799 10.1016/s0002-9394(03)00239-3

[18] Ayyala RS, Zurakowski D, Monshizadeh R, Hong CH, Richards D, Layden WE, et al Comparison of double-plate Molteno and Ahmed glaucoma valve in patients with advanced uncontrolled glaucoma. Ophthalmic Surg Lasers. 2002; 33: 94–101. 11942556

[19] Ou Y, Yu F, Law SK, Coleman AL, Caprioli J. Outcomes of Ahmed glaucoma valve implantation in children with primary congenital glaucoma. Arch Ophthalmol. 2009; 127: 1436–1441. doi: 10.1001/archophthalmol.2009.267 19901208 10.1001/archophthalmol.2009.267

[20] Heuer DK, Budenz D, Coleman A. Aqueous shunt tube erosion. J Glaucoma. 2001; 10: 493–496. 11740221 10.1097/00061198-200112000-00010

[21] Morad Y, Donaldson CE, Kim YM, Abdolell M, Levin AV. The Ahmed drainage implant in the treatment of pediatric glaucoma. Am J Ophthalmol. 2003; 135: 821–829. 12788122 10.1016/s0002-9394(02)02274-2

[22] Topouzis F, Coleman AL, Choplin N, Bethlem MM, Hill R, Yu F, et al Follow-up of the original cohort with the Ahmed glaucoma valve implant. Am J Ophthalmol. 1999; 128: 198–204. 10458176 10.1016/s0002-9394(99)00080-x

[23] Lee EK, Yun YJ, Lee JE, Yim JH, Kim CS. Changes in corneal endothelial cells after Ahmed glaucoma valve implantation: 2-year follow-up. Am J Ophthalmol. 2009; 148: 361–367. doi: 10.1016/j.ajo.2009.04.016 19505676 10.1016/j.ajo.2009.04.016

[24] Da Mata A, Burk SE, Netland PA, Baltatzis S, Christen W, Foster CS. Management of uveitic glaucoma with Ahmed glaucoma valve implantation. Ophthalmology. 1999; 106: 2168–2172. doi: 10.1016/S0161-6420(99)90500-6 10571354 10.1016/S0161-6420(99)90500-6

[25] Law SK, Nguyen A, Coleman AL, Caprioli J. Comparison of safety and efficacy between silicone and polypropylene Ahmed glaucoma valves in refractory glaucoma. Ophthalmology. 2005; 112: 1514–1520. doi: 10.1016/j.ophtha.2005.04.012 16005977 10.1016/j.ophtha.2005.04.012

[26] Ayyala RS, Harman LE, Michelini-Norris B, Ondrovic LE, Haller E, Margo CE, et al Comparison of different biomaterials for glaucoma drainage devices. Arch Ophthalmol. 1999; 117: 233–236. 10037569 10.1001/archopht.117.2.233

[27] Ayyala RS, Michelini-Norris B, Flores A, Haller E, Margo CE. Comparison of different biomaterials for glaucoma drainage devices: part 2. Arch Ophthalmol. 2000; 118: 1081–1084. 10922202 10.1001/archopht.118.8.1081

[28] Ishida K, Netland PA, Costa VP, Shiroma L, Khan B, Ahmed II. Comparison of polypropylene and silicone Ahmed Glaucoma Valves. Ophthalmology. 2006; 113: 1320–1326. doi: 10.1016/j.ophtha.2006.04.020 16877071 10.1016/j.ophtha.2006.04.020

[29] Gedde SJ, Lee RK. Comparing glaucoma drainage implants. Am J Ophthalmol. 2010; 149: 875–877. doi: 10.1016/j.ajo.2010.02.016 20510685 10.1016/j.ajo.2010.02.016

